# Case Report: Dental implant displacement into the contralateral nasal cavity managed by endoscopic retrieval

**DOI:** 10.3389/froh.2026.1843746

**Published:** 2026-05-20

**Authors:** Horatiu Urechescu, Cristiana Cuzic, Alisia Pricop, Marius Pricop

**Affiliations:** 1 Department of Oral and Maxillo-Facial Surgery, “Victor Babes” University of Medicine and Pharmacy, Timisoara, Romania; 2 Department of Prosthodontics, University of Medicine and Pharmacy “Victor Babes”, Timisoara, Romania; 3 TADERP Research Center—Advanced and Digital Techniques for Endodontic, Restorative and Prosthetic Treatment, University of Medicine and Pharmacy “Victor Babes”, Timisoara, Romania; 4 Research Center in Dental Medicine Using Conventional and Alternative Technologies, School of Dental Medicine, University of Medicine and Pharmacy “Victor Babes”, Timisoara, Romania

**Keywords:** anterior maxilla, CBCT, contralateral displacement, dental implant displacement, endoscopic retrieval, implant complications, nasal cavity

## Abstract

Dental implant placement in the anterior maxilla requires precise anatomical assessment and strict control of surgical technique due to the proximity of the nasal cavity and maxillary sinuses. Although implant displacement into adjacent structures has been reported, involvement of the nasal cavity remains rare. We report the case of a male patient who developed intraoperative displacement of a dental implant into the contralateral nasal cavity during full-arch maxillary implant placement. The patient presented one day postoperatively and remained asymptomatic. Clinical examination and anterior rhinoscopy were inconclusive. CBCT revealed the implant crossing the midline and perforating the nasal mucosa, findings subsequently confirmed by nasal endoscopy, which localized the implant within the inferior meatus of the left nasal cavity. The exact cause of displacement could not be objectively determined due to the lack of complete intraoperative documentation from the referring institution. However, CBCT findings and clinical context may suggest a possible multifactorial mechanism; this interpretation remains hypothetical and may involve reduced anterior maxillary bone volume, particularly in terms of ridge width, combined with altered control of insertion dynamics, potentially leading to sudden loss of resistance and unintended apical advancement of the implant. The implant was successfully removed using an endoscopic-assisted transnasal approach under general anesthesia, allowing direct visualization and controlled retrieval. The postoperative course was uneventful. This case highlights the importance of thorough preoperative planning, accurate radiological assessment, and careful control of surgical technique in anatomically challenging regions. Early diagnosis using CBCT and endoscopic evaluation is essential, while endoscopic management represents a safe and effective approach for implant retrieval from the nasal cavity.

## 1 Introduction

Dental implant placement in the anterior maxilla requires precise preoperative planning due to the close proximity of critical anatomical structures such as the maxillary sinuses and the nasal cavity. Accurate assessment of bone morphology and available bone volume is essential to ensure proper implant positioning and to achieve primary stability (1–3).

Imaging plays a central role in treatment planning. While panoramic radiography is frequently used for initial assessment, its two-dimensional nature limits accurate evaluation of buccopalatal bone dimensions and spatial relationships (4, 5). Cone-beam computed tomography (CBCT) provides a three-dimensional assessment of anatomical structures and is considered essential in cases with reduced bone volume or proximity to vital structures (2, 3).

Implant displacement into adjacent craniofacial structures is a recognized complication, most commonly involving the maxillary sinus (6, 7). However, displacement into the nasal cavity is considerably less frequent and remains poorly documented in the literature (8, 9). Such complications may occur when anatomical limitations are underestimated or when surgical control during implant insertion is insufficient, particularly in anatomically compromised sites.

We report a rare case of intraoperative displacement of a dental implant into the contralateral nasal cavity, successfully managed using an endoscopic-assisted transnasal approach. The case highlights the importance of accurate preoperative assessment, appropriate surgical technique, and early diagnosis using advanced imaging.

## 2 Case description

A male patient in his early 50s was referred to the Department of Oral and Maxillofacial Surgery one day after undergoing full-arch maxillary implant placement in a private dental clinic. The patient had been edentulous in the maxillary arch for approximately six months following multiple tooth extractions.

During the initial procedure, several implants were placed in the maxilla. However, the implant intended for the right lateral incisor region was inadvertently displaced beyond the alveolar bone during insertion. The referring clinician was unable to retrieve the implant using a crestal approach and subsequently referred the patient for further management.

No intraoperative radiographic verification of implant position was performed.

Additional procedural details were obtained retrospectively from the referring clinician. The displaced implant was a MIS SEVEN (MIS Implants Technologies Ltd., Shlomi, Israel) with a diameter of 3.75 mm and a length of 10 mm. Osteotomy preparation was performed using a surgical motor set at approximately 1,000 rpm and 55 Ncm torque. Implant insertion was subsequently carried out using the same motor-driven approach, without modification of rotational speed or torque settings.

At the time of presentation, the patient was systemically healthy, with no relevant medical history. He reported no pain, bleeding, or neurosensory disturbances. Clinical examination revealed mild localized edema in the anterior maxillary region, with intact mucosa and no signs of dehiscence, suppuration, or ecchymosis. No oro-nasal or oro-antral communication was detected.

Anterior rhinoscopy did not reveal any visible foreign body or mucosal abnormality. However, this finding was considered consistent with the limited field of view of anterior rhinoscopy, particularly for foreign bodies located posteriorly or superiorly within the nasal cavity.

Clinical photographs at the time of presentation were not available, as the patient was referred from an external clinic and no standardized photographic documentation had been performed prior to referral.

### 2.1 Diagnostic assessment

**Figure 1:**
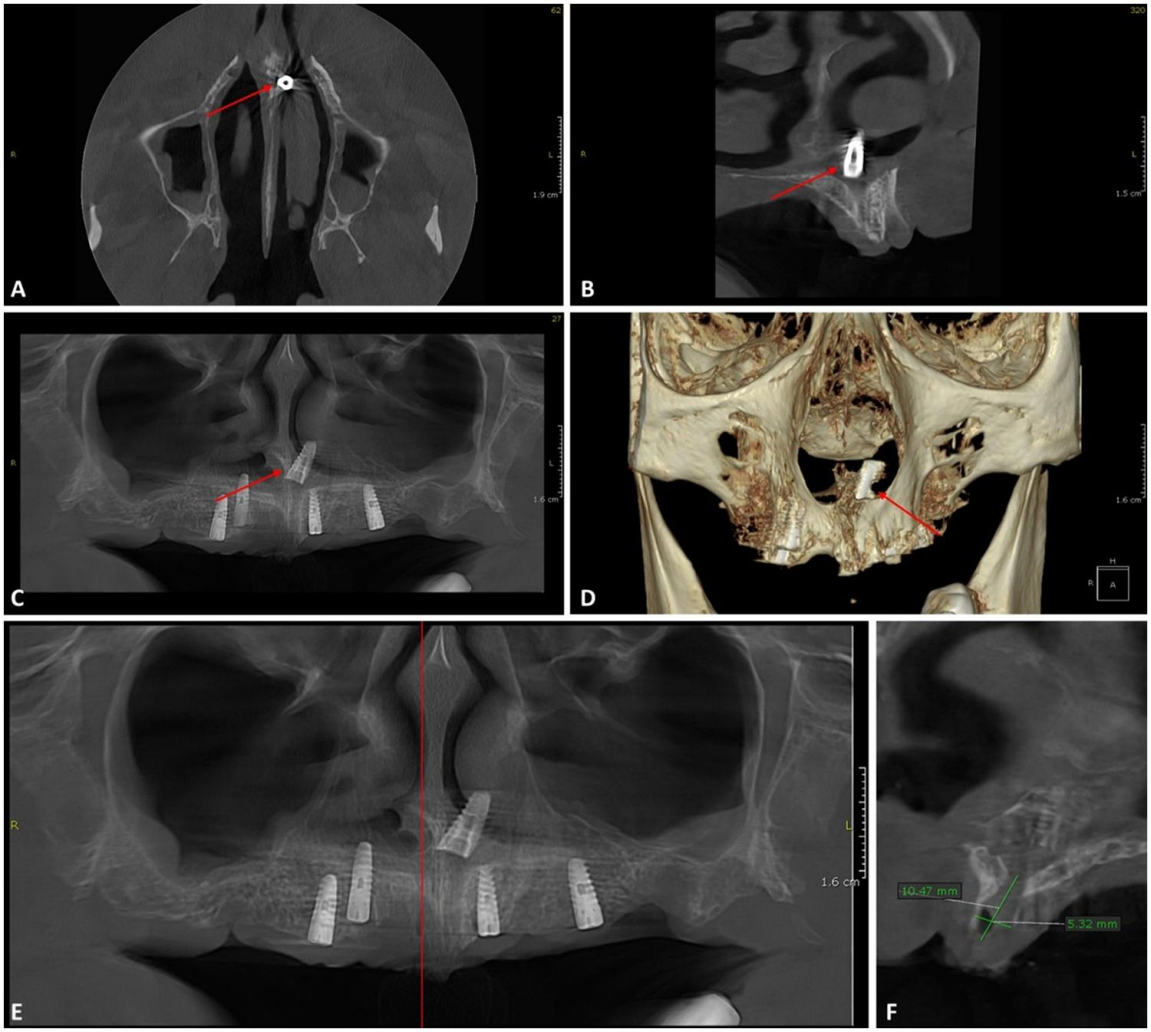
CBCT images showing the displaced dental implant in the left nasal cavity (red arrow): **(A)** axial view—implant crossing the midline and perforating the nasal floor. **(B)** Sagittal view—implant apex penetrating the nasal mucosa. **(C)** Coronal view—implant misalignment relative to adjacent implants. **(D)** 3D reconstruction—visualization of the implant within the nasal cavity. **(E)** Panoramic reconstruction indicating the section plane used for quantitative assessment (red vertical line). **(F)** Cross-sectional view at the level indicated in **(E)**, demonstrating reduced residual ridge height (∼10.5 mm) and buccopalatal width (∼5.3 mm) at the implant site.

CBCT was performed to determine the exact position of the displaced implant and its relationship to surrounding anatomical structures. The imaging revealed that the implant, initially intended for the right lateral incisor region, had been displaced across the midline and was located within the left nasal cavity, perforating the nasal floor mucosa. The implant was positioned in a near-vertical orientation, with its apex protruding into the nasal cavity. CBCT also demonstrated reduced anterior maxillary bone volume, with a thin residual alveolar ridge at the intended implant site. Quantitative assessment at this level showed a residual ridge height of approximately 10.5 mm and a buccopalatal width of approximately 5.3 mm. Additionally, other implants placed during the same procedure were found in close proximity to the maxillary sinus and nasal floor, with at least one implant in the anterior region positioned notably superior relative to adjacent anatomical structures (Figure 1).

Mucosal thickening suggestive of inflammatory changes was observed in the left nasal cavity as well as in both maxillary sinuses.

**Figure 2:**
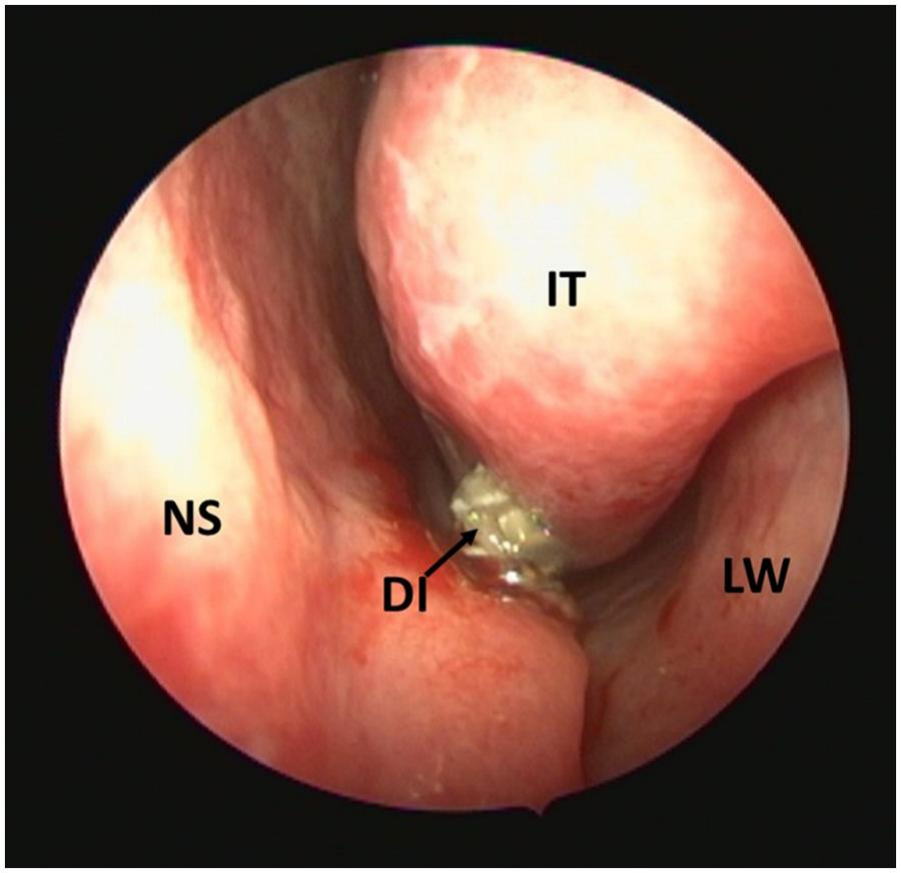
Endoscopic view of the left nasal cavity showing the displaced implant (DI) located in the inferior meatus, beneath the inferior turbinate (IT), in close proximity to the nasal septum (NS) and lateral nasal wall (LW).

To further evaluate the nasal cavity, nasal endoscopy was performed. This confirmed the presence of the implant within the left nasal cavity, with visible perforation of the nasal mucosa (Figure 2).

Endoscopic evaluation localized the exposed portion of the implant within the left inferior meatus, beneath the inferior turbinate and in close proximity to the nasal septum and lateral nasal wall. The implant appeared partially embedded in the mucosa of the nasal floor rather than freely mobile within the nasal cavity. This anatomical position may explain the absence of nasal obstruction, epistaxis, pain, or foreign-body sensation at presentation, as the implant did not appear to significantly compromise the main nasal airflow pathway and was not associated with active bleeding or purulent discharge. The chronological sequence of the diagnostic and therapeutic management is summarized in Table 1.

**Table 1:** Timeline of clinical events and management.

**Time Point**	**Event**
∼6 months before presentation	Multiple maxillary tooth extractions performed; patient became fully edentulous in the maxilla
Day 0 (initial surgery)	Full-arch maxillary implant placement performed in a private dental clinic
Day 0 (intraoperative)	Implant intended for the right lateral incisor region displaced beyond alveolar bone during insertion; unsuccessful attempt at crestal retrieval
Day 1 (presentation)	Patient referred to Oral and Maxillofacial Surgery department; asymptomatic with mild anterior maxillary edema
Day 1	Clinical examination and anterior rhinoscopy performed (no visible foreign body detected)
Day 1	CBCT imaging revealed implant displaced into the left nasal cavity, crossing the midline
Day 1	Nasal endoscopy confirmed implant perforating the nasal mucosa
Day 2 (treatment)	Endoscopic-assisted transnasal removal performed under general anesthesia
Immediate postoperative period	Uneventful recovery; mild discomfort and nasal congestion managed conservatively
1-week follow-up	Complete resolution of symptoms; no infection or complications observed
Subsequent follow-up	Stable healing; no functional or aesthetic complaints; future implant rehabilitation postponed

### 2.2 Therapeutic intervention

Based on the imaging and endoscopic findings, a decision was made to remove the displaced implant using an endoscopic-assisted transnasal approach under general anesthesia.

The selection of an endoscopic-assisted transnasal approach was based on the implant's location within the nasal cavity and its accessibility via a minimally invasive route. This approach was preferred in order to avoid more invasive procedures, such as transoral or Caldwell–Luc techniques, which are associated with increased surgical morbidity and reduced direct visualization of the nasal cavity.

The patient was positioned supine, and airway protection was ensured through endotracheal intubation. Given the implant's position within the superior aspect of the nasal cavity and the risk of posterior displacement into the nasopharynx, airway protection through endotracheal intubation was considered essential to prevent aspiration during manipulation. A 30-degree rigid nasal endoscope was used to provide optimal visualization of the nasal cavity. Continuous suction was maintained using a nasal aspirator to ensure a clear operative field and reduce the risk of aspiration.

**Figure 3:**
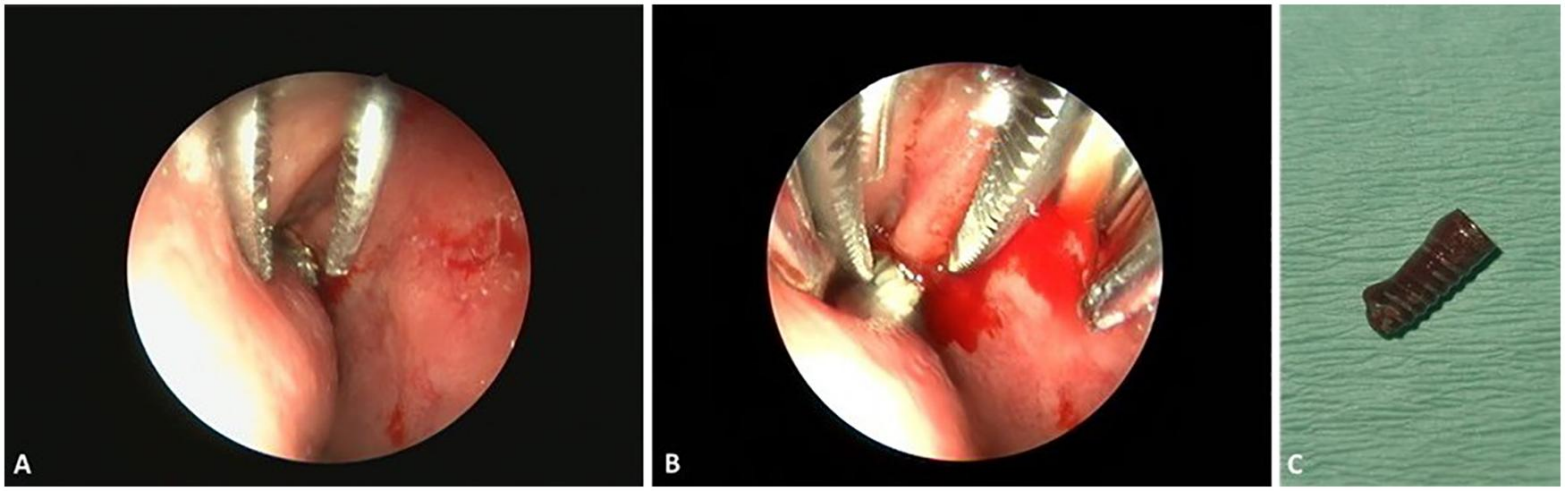
Endoscopic-assisted removal of the displaced dental implant from the left nasal cavity: **(A)** implant grasped with a hemostasis clamp under endoscopic visualization. **(B)** Controlled extraction through the nasal passage with visible mucosal bleeding. **(C)** Retrieved dental implant following successful removal.

Endoscopic examination identified the implant partially embedded in the nasal mucosa. A hemostatic forceps was introduced under direct visualization, and the exposed portion of the implant was carefully grasped (Figure 3A). Controlled traction was applied along the long axis of the implant to disengage it from surrounding tissues.

Particular attention was paid to prevent posterior displacement of the implant into the nasopharynx, which could have posed a risk of aspiration. Continuous visualization and controlled manipulation were maintained throughout the procedure.

Moderate mucosal bleeding occurred during extraction and was effectively controlled using local compression and suction (Figure 3B).

The implant was successfully removed in a single attempt without fragmentation or further displacement (Figure 3C). Final endoscopic inspection confirmed the absence of residual foreign material and preserved integrity of the surrounding mucosa.

### 2.3 Follow-Up and outcomes

The immediate postoperative course was uneventful. The patient experienced mild discomfort and nasal congestion, which resolved within 48 h with conservative management, including analgesics, saline nasal irrigation, and short-term use of nasal decongestants. Antibiotic prophylaxis was prescribed to reduce the risk of secondary infection.

At one-week follow-up, complete resolution of nasal bleeding and edema was observed, with no evidence of infection or persistent nasal symptoms. Continued follow-up over a period of 1 month confirmed satisfactory healing, with preservation of normal nasal breathing and no functional or aesthetic complaints.

No adverse or unanticipated events were observed during the follow-up period, and the patient demonstrated good adherence to postoperative recommendations. Future implant rehabilitation was postponed pending complete bone healing and reassessment of the anatomical site.

## 3 Discussion

This case represents an instance of intraoperative displacement of a dental implant into the contralateral nasal cavity, rather than true postoperative migration. Most reports in the literature describe implant migration as a delayed phenomenon, often associated with progressive bone resorption, inflammatory processes, or inadequate primary stability (10–13). In contrast, the short interval between implant placement and diagnosis in the present case strongly supports that displacement occurred during insertion. The present case is further notable due to the contralateral displacement across the midline and the immediate postoperative detection, features that are rarely reported.

Implant displacement into adjacent craniofacial structures has been well documented, most frequently involving the maxillary sinus (6, 7). However, involvement of the nasal cavity is considerably less common and remains sparsely reported (8, 9). In this context, understanding the potential mechanisms of intraoperative displacement is essential for both prevention and early recognition.

The mechanism of displacement in the present case is likely multifactorial and should be interpreted as hypothetical, based on retrospective imaging findings and limited procedural data. From an anatomical perspective, reduced anterior maxillary bone volume may have resulted in decreased mechanical resistance during implant insertion, predisposing to loss of primary stability. In addition, the anatomical configuration of the anterior nasal floor may have contributed. In edentulous patients, the bony nasal septum at this level may be thin, attenuated, or partially absent, potentially allowing communication between the two sides of the nasal cavity. CBCT findings suggest that the implant trajectory deviated from the intended vertical axis, progressing toward and through the nasal floor. Once the cortical boundary was perforated, the lack of resistance within the nasal cavity may have allowed further advancement along a path of least resistance, potentially explaining the contralateral displacement. However, the exact trajectory cannot be definitively reconstructed and should be interpreted with caution.

In addition to anatomical considerations, technical factors may have played a contributory role. Excessive insertion forces, inadequate control of rotational speed, or over-preparation of the osteotomy have been described as possible contributing factors in implant displacement (10, 11). In the present case, both osteotomy preparation and implant insertion were performed using motor-driven settings (approximately 1,000 rpm and 55 Ncm torque) without intraoperative adjustment. Under such conditions, reduced tactile feedback may delay recognition of loss of resistance during insertion. In the presence of limited bone support, this may facilitate uncontrolled apical advancement of the implant. Nevertheless, due to the absence of complete intraoperative records, including real-time variations in insertion parameters and surgical decision-making, these observations cannot be considered independently causative.

From a clinical perspective, early recognition of intraoperative warning signs is essential, particularly in anatomically compromised anterior maxillae. Indicators such as sudden loss of resistance, unexpected rapid apical advancement, absence of progressive torque increase, and reduced tactile feedback should raise suspicion of compromised control during implant placement. In such situations, implant insertion should be immediately interrupted and the position reassessed. If primary stability is not achieved or the implant trajectory becomes uncertain, further advancement should be avoided.

In cases of reduced anterior maxillary bone volume, a more conservative and staged approach may be indicated. This may include ridge augmentation procedures, the use of shorter implants, or guided implant placement to better control angulation and depth. Careful preoperative planning combined with conservative intraoperative decision-making is essential to minimize the risk of displacement into adjacent anatomical structures.

An important diagnostic aspect in this case was the discrepancy between clinical examination and imaging findings. Despite the implant being located within the nasal cavity, anterior rhinoscopy failed to detect the foreign body, a limitation that has been previously described (14). In contrast, CBCT allows accurate three-dimensional localization, while nasal endoscopy provides direct visualization and confirmation of mucosal involvement (13). These complementary modalities are therefore essential in suspected cases of implant displacement.

Regarding management, several surgical approaches have been described depending on the location of the displaced implant. While transoral or Caldwell–Luc approaches have traditionally been used for maxillary sinus retrieval, endoscopic techniques have gained increasing popularity due to their minimally invasive nature and improved visualization (6, 13). In the present case, the endoscopic-assisted transnasal approach allowed precise localization and controlled removal without the need for additional surgical access, resulting in minimal morbidity.

However, this technique requires careful execution. One of the main intraoperative risks is posterior displacement of the implant into the nasopharynx, which may result in aspiration. For this reason, strict airway protection, continuous endoscopic visualization, and controlled manipulation are essential. In the present case, the use of general anesthesia with endotracheal intubation, combined with continuous suction and endoscopic guidance, ensured safe and effective retrieval.

A limitation of this report is the absence of complete intraoperative documentation from the referring institution. Although certain procedural details were obtained retrospectively, the lack of comprehensive operative records limits accurate reconstruction of the surgical sequence. Consequently, it is not possible to definitively distinguish between anatomical and technical contributing factors, and any interpretation of the displacement mechanism must remain inferential.

Overall, this case underscores the importance of thorough anatomical assessment, accurate radiological evaluation, particularly using CBCT, and careful control of surgical technique in anatomically challenging regions. Early diagnosis using CBCT and endoscopic evaluation, combined with appropriate surgical management, is essential to ensure safe and predictable outcomes.

## 4 Conclusion

Intraoperative displacement of dental implants into the nasal cavity is a rare but clinically significant complication that requires prompt recognition and management. This case highlights the importance of thorough preoperative assessment of anatomical limitations, particularly in edentulous patients with reduced anterior maxillary bone volume.

Accurate diagnosis relies on advanced imaging modalities such as CBCT, especially when clinical examination is inconclusive. Endoscopic-assisted transnasal retrieval represents a safe and minimally invasive approach, allowing direct visualization and controlled removal of displaced implants.

Although the exact mechanism of displacement cannot be definitively established, this case supports a possible multifactorial interaction between anatomical constraints and technical factors. Careful control of surgical technique, including insertion parameters, implant angulation, and depth control, remains essential to minimize the risk of such complications. In anatomically compromised anterior maxillae, early recognition of intraoperative warning signs and conservative decision-making may help prevent unintended displacement and improve clinical outcomes.

## 5 Patient perspective

The patient reported no significant symptoms at the time of presentation and was initially unaware of the extent of the complication. After being informed about the diagnosis and the need for surgical removal, he expressed concern about the presence of the implant in the nasal cavity and the potential risks involved.

Following the procedure, the patient reported only mild discomfort and nasal congestion, which resolved within a few days. He expressed satisfaction with the minimally invasive nature of the treatment and the rapid recovery.

At follow-up, the patient remained asymptomatic and reported no functional or aesthetic concerns. He was informed about the need for careful reassessment before future implant rehabilitation and expressed understanding and agreement with the proposed management plan.

## Data Availability

The raw data supporting the conclusions of this article will be made available by the authors, without undue reservation.
